# Radiological Insights From Two Distinct Cases of Pineal Region Meningioma: A Case Report

**DOI:** 10.7759/cureus.57796

**Published:** 2024-04-07

**Authors:** Chakradhar Ravipati, Michael Antony Vikram, Karthik Krishna Ramakrishnan, Anbalagan Malaichamy

**Affiliations:** 1 Radiodiagnosis, Saveetha Medical College and Hospital, Saveetha Institute of Medical and Technical Sciences (SIMATS) Saveetha University, Chennai, IND

**Keywords:** parinaud syndrome, brain tumor, magnetic resonance imaging, pineal region, meningioma

## Abstract

This case report delineates the radiological evaluation and diagnostic intricacies of two unique cases of pineal region meningioma, underscoring the pivotal role of advanced imaging techniques in the accurate diagnosis and management of such rare tumors. Pineal region tumors represent a heterogeneous group of neoplasms, with meningiomas in this location being particularly uncommon, thereby posing significant challenges in diagnosis and therapeutic decision-making. The first case involves a 40-year-old female presenting with progressive headaches and visual disturbances with symptoms of increased intracranial pressure, whereas the second case describes a 30-year-old male presenting with headache, dizziness, difficulty with balance, and cognitive decline. Both patients underwent a comprehensive diagnostic workup, including magnetic resonance imaging (MRI), which revealed tumors in the pineal region exhibiting characteristics suggestive of meningioma. The MRI findings in both cases included well-defined mass lesions showing iso- to hypointense signal on T1-weighted images with robust contrast enhancement. Additionally, the radiological assessment was instrumental in differentiating these meningiomas from other pineal region tumors, such as germinomas or pineocytomas, based on their distinctive imaging features and the absence of dissemination. Surgical resection followed by histopathological examination confirmed the diagnosis of meningioma in both cases. This report highlights the critical role of radiological imaging in the early detection and differentiation of pineal region tumors, emphasizing the need for a multidisciplinary approach to achieve optimal patient outcomes.

## Introduction

Meningiomas are the most common primary intracranial tumors, accounting for approximately 13-26% of all intracranial tumors [1]. They are typically benign and slow-growing tumors that arise from the meninges, the protective membranes surrounding the brain and spinal cord. While they can occur in various locations within the central nervous system, meningiomas arising in the pineal region are relatively rare.

Pineal region tumors are rare intracranial tumors that can present with a variety of symptoms and radiological findings. These tumors, which account for 0.5-1.6% of all intracranial tumors [2], can be challenging to diagnose and differentiate from each other and include germ cell tumors, glial tumors, pineal parenchymal tumors, meningiomas, cysts, and lymphomas. Furthermore, the radiological features of these tumors vary, with different signal characteristics and enhancement patterns on magnetic resonance imaging (MRI) [3]. These features, along with the relationship of the tumor to surrounding structures such as the posterior third ventricle, Sylvian aqueduct, vein of Galen, and tentorium, can provide valuable insights in the evaluation and differential diagnosis of pineal region tumors [4].

In this case series, we present two cases of pineal region meningioma with a focus on their unique radiological features differentiating them from other pineal region pathologies. Furthermore, we discuss the implications of these radiological insights for the evaluation, diagnosis, and management of pineal region tumors.

## Case presentation

Case presentation 1

A 40-year-old female presented to the neurology department with a three-month history of progressively worsening headaches, intermittent blurred vision, and episodes of confusion. Ophthalmological examination revealed mild bilateral papilledema, suggestive of increased intracranial pressure, and neurological examination revealed an upward gaze palsy and pupillary hyporeflexia, suggestive of Parinaud syndrome which is indicative of dorsal midbrain compression in the pineal region.

**Figure 1:**
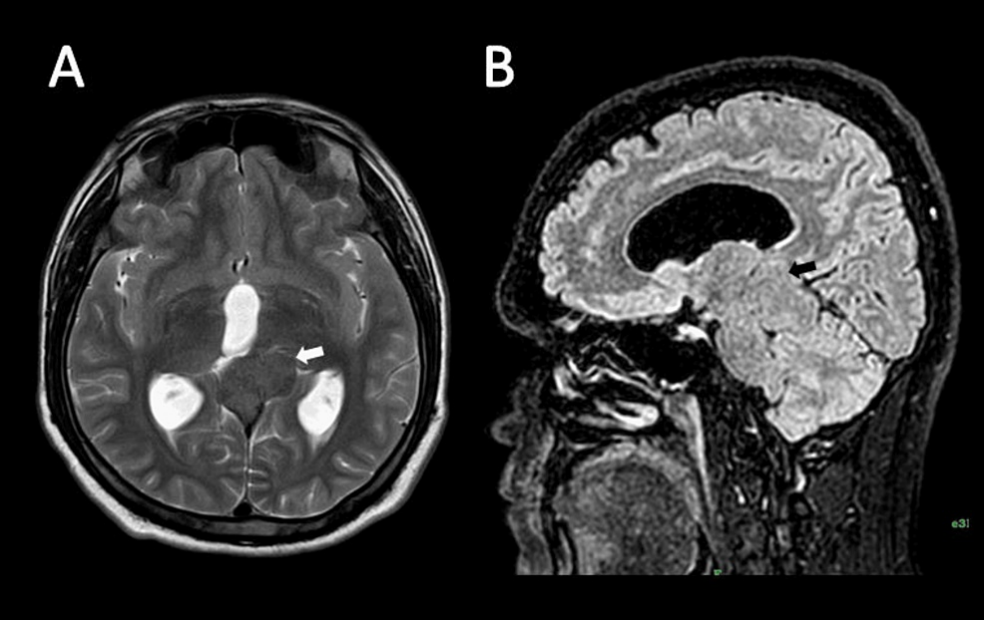
Case 1, a 40-year-old woman. T2-weighted and FLAIR sequences. (A) Axial T2-weighted image shows a well-defined, lobulated, extra-axial, pineal region mass isointense to mildly hypointense relative to gray matter. This lesion causes a mass effect on the dorsomedial aspect of the left thalamus (white arrow). (B) The sagittal FLAIR image shows this lesion (black arrow) is mildly hypointense to gray matter. FLAIR: fluid-attenuated inversion recovery

**Figure 2:**
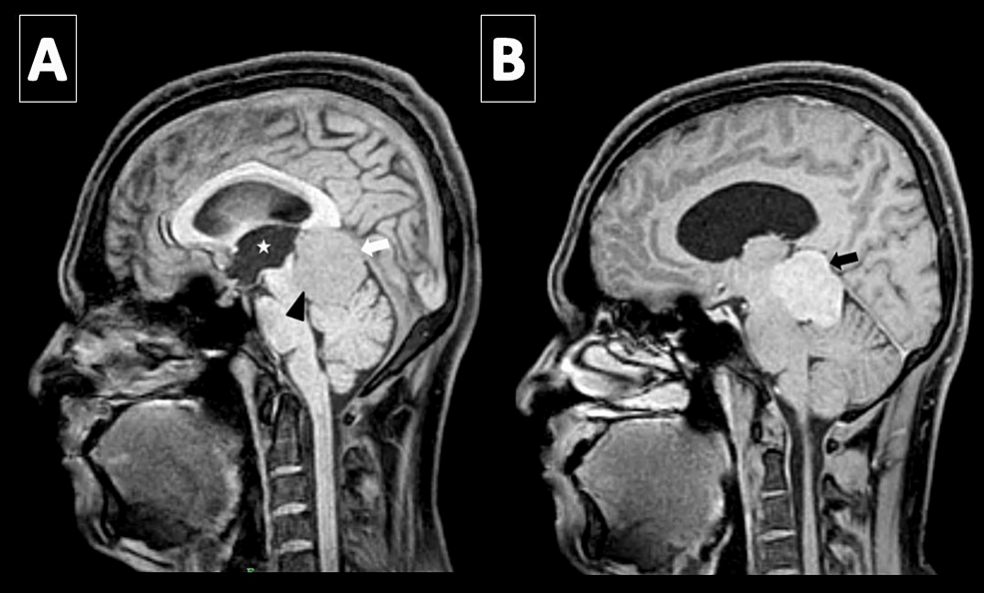
Case 1, a 40-year-old woman. Precontrast and postcontrast T1-weighted fat-suppressed sequences. (A) The sagittal precontrast T1-weighted image shows the infratentorial pineal region mass (white arrow) is isointense to gray matter and causes a mass effect on the tectal plate (black arrowhead) and cerebral aqueduct, resulting in mild obstructive upstream dilatation of the third ventricle (white star). (B) The sagittal postcontrast T1-weighted image shows the lesion homogeneously enhances (arrow).

**Figure 3:**
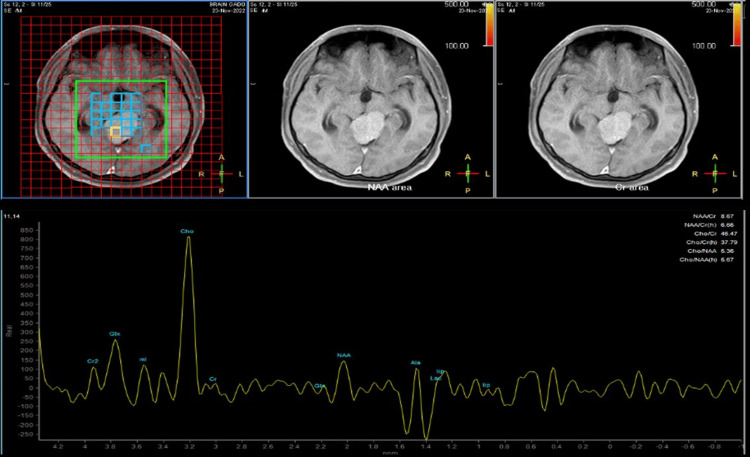
Case 1, a 40-year-old woman. MR spectroscopy. Multi-voxel MR spectroscopy reveals that the lesion has a markedly elevated choline peak and a reduced NAA peak. NAA: N-acetylaspartate

MRI of the brain with and without contrast revealed a solitary well-defined, lobulated, extra-axial, infratentorial, non-diffusion-restricted mass lesion measuring ~3.7×3.5 cm with epicenter in the pineal region. This lesion appeared isointense to mildly hypointense on T2-weighted and fluid-attenuated inversion recovery (FLAIR) images (Figure 1A, 1B) and isointense to gray matter on T1-weighted image (Figure 2A). No significant parenchymal edema was noted. This lesion showed intense homogeneous enhancement (Figure 2B). The lesion compressed the tectal plate of the midbrain and aqueduct of Sylvius, resulting in obstructive hydrocephalus and compression of the left thalamus (Figure 1B, Figure 2A). No evidence of necrosis, cystic degeneration, or hemorrhage was noted within the lesion. MR spectroscopy of the lesion showed a markedly elevated choline peak and reduced N-acetylaspartate (NAA), suggestive of a non-neuronal neoplasm given the other imaging findings (Figure 3).

**Figure 4:**
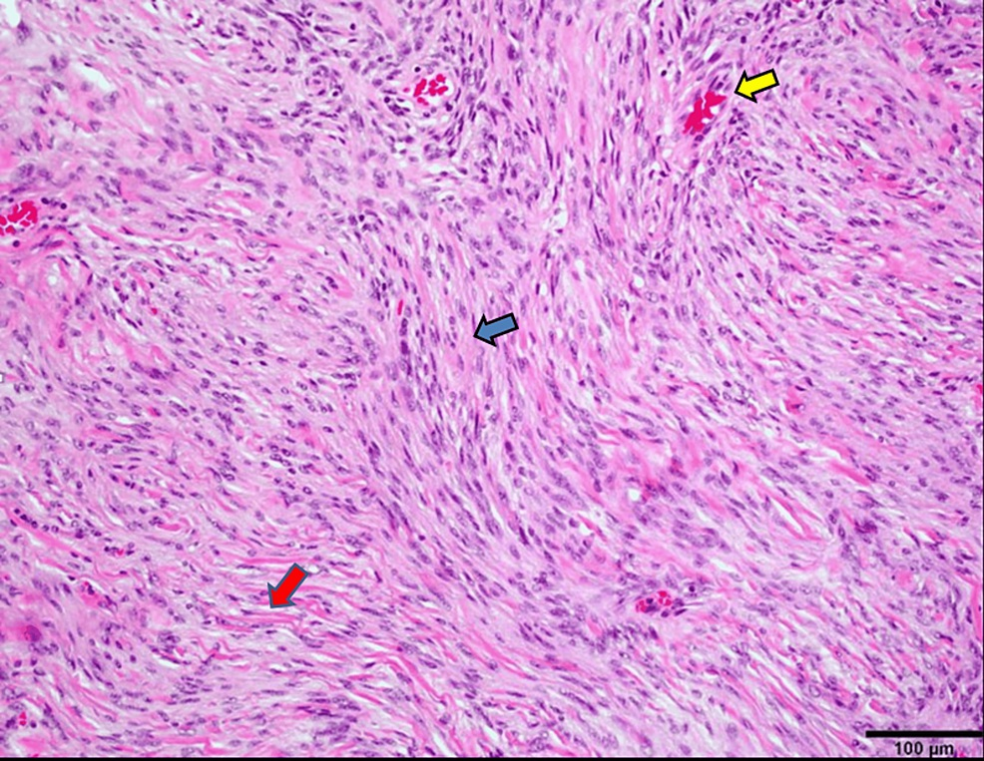
Case 1, a 40-year-old woman. Histopathology. Hematoxylin and eosin stain of the resected mass showed a cellular neoplasm composed of spindle cells (blue arrow) with elongated and bland nuclei arranged in fascicles interspread by collagen (red arrow) and blood vessels (yellow arrow), without cellular whorls or meningothelial cells. No mitosis, necrosis, or atypia was seen. These features were consistent with a WHO grade 1 fibroblastic meningioma.

The patient underwent a suboccipital craniotomy and gross total resection of the tumor which was confirmed to be a WHO grade 1 fibroblastic meningioma as shown in Figure 4. During the initial six-month follow-up period, repeated MRI scans showed no evidence of tumor recurrence. The improvement in the patient's preoperative symptoms, such as headaches and visual disturbances, significantly enhanced their quality of life. Neurological examinations conducted at subsequent follow-up visits at six and 12 months post-surgery consistently revealed normal findings, with no signs of neurological deficits. The patient reported a return to her daily activities without any limitations.

Case presentation 2

**Figure 5:**
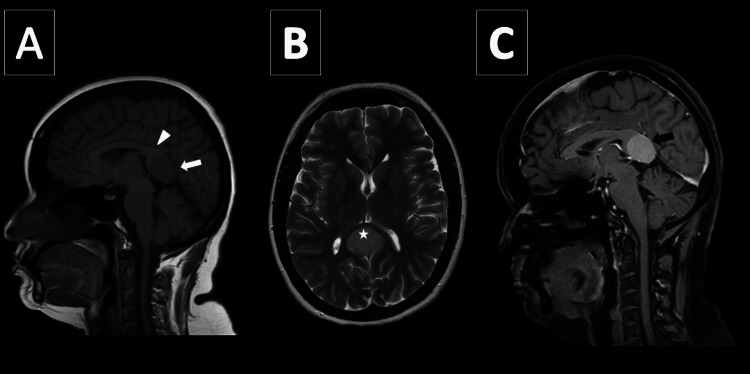
Case 2, a 30-year-old man. T2-weighted and pre- and postcontrast T1-weighted sequences. (A) The sagittal precontrast T1-weighted image shows a well-defined, extra-axial, supratentorial mass centered in the pineal region (arrow) that is isointense to gray matter and compresses the splenium of the corpus callosum (arrowhead). (B) The lesion is mildly hyperintense on T2-weighted images. (C) The sagittal postcontrast fat-suppressed T1-weighted image shows the mass homogeneously enhances.

A 30-year-old male patient initially presented to the neurology department with symptoms of headache, dizziness, difficulty with balance, and cognitive decline over several months. Upon further investigation, MRI revealed a solitary well-defined, extra-axial, supratentorial, non-diffusion-restricted lesion, centered in the pineal region. This lesion appeared isointense to gray matter on T1-weighted imaging (Figure 5A) and mildly hyperintense to gray matter on T2-weighted imaging (Figure 5B). This lesion compressed the splenium of the corpus callosum antero-superiorly and abutted the precuneus bilaterally. No significant parenchymal edema was noted. The postcontrast imaging of this lesion showed intense homogeneous enhancement (Figure 5C). No necrosis, cystic degeneration, hemorrhagic, or calcification was seen within the lesion. MR spectroscopy was unremarkable.

**Figure 6:**
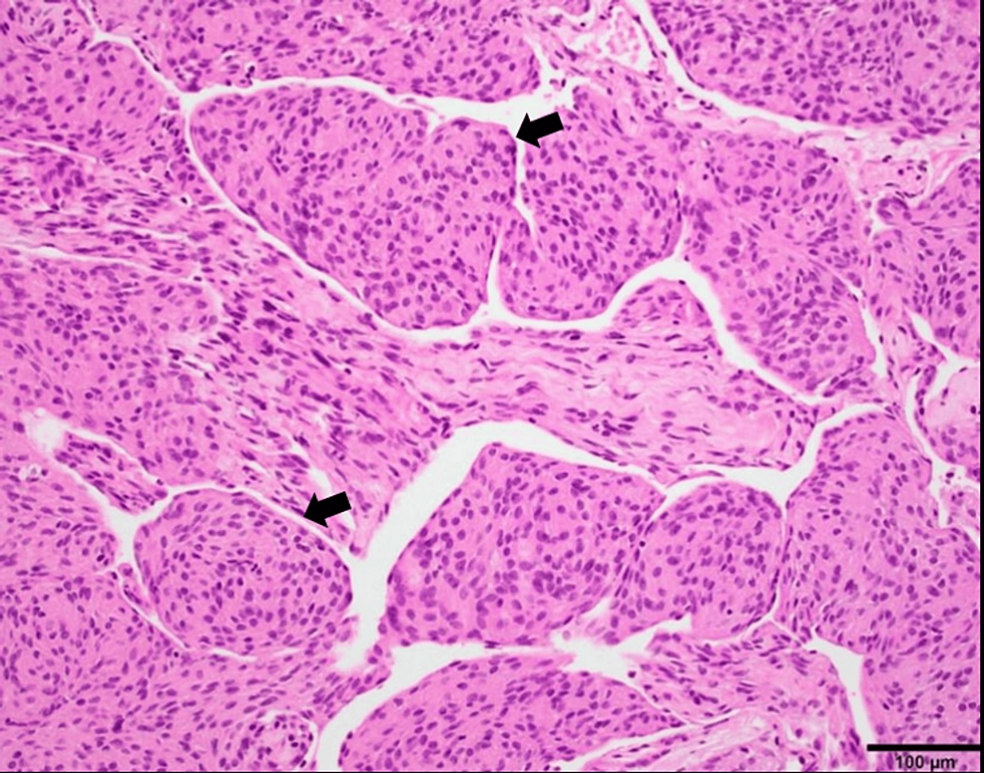
Case 2, a 30-year-old man. Histopathology. Hematoxylin and eosin stain of the partially resected mass shows fragments of tumor composed of ovoid to spindle-shaped meningothelial cells arranged in vague intersecting fascicles with occasional whorl formation (arrows). These tumor cells have fine nuclear chromatin and moderate eosinophilic cytoplasm. There was no evidence of increased cellularity, mitotic activity, nuclear pleomorphism, or necrosis. These features were consistent with a WHO grade 1 meningothelial meningioma.

The patient underwent a minimally invasive endoscopic resection of the pineal mass, which histopathological examination revealed was a WHO grade 1 meningothelial meningioma as shown in Figure 6, and immunohistochemistry was negative for progesterone receptor expression and Ki-67 elevation. The endoscopic approach facilitated a significant reduction in tumor mass and alleviated some of the patient's initial symptoms, such as severe headaches. However, during the follow-up visits at three, six, and 12 months post-surgery, although MRI scans confirmed that there was no tumor recurrence, the patient continued to experience mild cognitive impairments and occasional sleep disturbances. Despite the challenges, the patient reported an improvement in overall quality of life compared to the preoperative state but also acknowledged the ongoing need for supportive care and rehabilitation to manage the residual symptoms. 

## Discussion

Pineal region masses encompass a diverse spectrum of lesions that pose diagnostic challenges to clinicians and radiologists. These masses, located in the pineal gland or adjacent structures, can manifest with various clinical presentations, including headaches, visual disturbances, endocrine dysfunction, and neurologic deficits. Differential diagnosis of pineal region masses is broad and includes neoplastic and non-neoplastic entities. Non-neoplastic lesions of the pineal region encompass a wide range of entities, including pineal cysts, cystic lesions, and vascular abnormalities. Pineal cysts are among the most common incidental findings, often presenting as benign lesions [5]. Neoplastic lesions commonly encountered in the pineal region include pineal parenchymal tumors, such as pineocytomas and pineoblastomas, as well as germ cell tumors like germinomas, non-germinomatous germ cell tumors (NGGCTs), and teratomas [6]. Pineal region meningiomas, though rare, are tumors originating from the meninges and can arise from various locations such as the tela choroidea, velum interpositum, or falcotentorial junction [5]. These tumors are characterized by their cellular nature, and few of the cases exhibit calcifications within the lesion.

The reported cases provide insight into the uncommon and challenging condition of pineal region meningiomas. The intricacies involved in diagnosing and managing meningiomas in the pineal region underscore the complexity of neuro-oncology and highlight the need for advanced diagnostic tools and multidisciplinary approaches. Pineal region meningiomas, while rare, present significant challenges due to their unique location and the potential for differential diagnosis with other pineal gland tumors. Imaging is paramount to the identification and characterization of these tumors, providing essential clues about their nature and guiding therapeutic decisions.

On MRI, meningiomas demonstrate low to intermediate signal on T1-weighted images and intermediate to slightly high signal on T2-weighted images, with pronounced enhancement following contrast administration [5]. Advanced MRI techniques, including diffusion-weighted imaging (DWI) and perfusion-weighted imaging (PWI), may also be helpful in distinguishing meningiomas from other pineal region tumors by evaluating tumor cellularity and vascularity [7,8]. Furthermore, MR spectroscopy offers additional insights by analyzing the chemical composition of tumors, aiding in the differentiation of meningiomas from other neoplasms based on their metabolic profiles [9]. The use of these imaging modalities in conjunction with conventional MRI findings enriches the diagnostic process, enabling a more accurate assessment of tumor characteristics and behavior.

The treatment of pineal region meningiomas often involves surgical resection, which aims to remove the tumor while minimizing damage to surrounding brain structures. The choice of surgical approach, whether open surgery or minimally invasive techniques, depends on the tumor's size and location and the presence of surrounding vital structures. Recent advancements in neurosurgical techniques, including neuro-navigation and endoscopic surgery, have significantly improved the safety and efficacy of these procedures, allowing for more precise tumor resection with reduced postoperative complications [10,11].

Adjuvant therapies, such as radiation therapy and chemotherapy, are considered in cases where complete resection is not feasible or when the risk of recurrence is high. Stereotactic radiosurgery (SRS) and fractionated stereotactic radiotherapy (FSRT) have emerged as effective treatments for meningiomas, delivering high-dose radiation to the tumor while sparing adjacent healthy tissue [12,13]. Chemotherapy, while less commonly used for meningiomas due to their typically slow growth and benign nature, may be considered in aggressive or recurrent cases, with ongoing research into targeted therapies and immunotherapy offering new avenues for treatment [14,15].

The management of pineal region meningiomas also highlights the importance of a multidisciplinary team approach, involving neurosurgeons, radiologists, oncologists, and other specialists. This collaborative effort ensures comprehensive care, from accurate diagnosis through tailored treatment plans to postoperative follow-up and rehabilitation, ultimately aiming to maximize patient outcomes and quality of life [16].

## Conclusions

The presented cases of pineal region meningioma underscore the critical role of advanced imaging techniques, particularly MRI and MR spectroscopy, in narrowing the diagnosis. These modalities provided invaluable insights into tumor characteristics, aiding in surgical planning and confirmation of diagnosis post-surgery. While emphasizing the significance of expert surgical management, this report highlights the nuanced nature of recovery following interventions for pineal region meningiomas. While minimally invasive techniques offer benefits, complete symptom resolution may not always be attainable. However, meticulous surgical care, informed by precise imaging, facilitates significant improvements and maintains a high quality of life for patients. These insights underscore the importance of tailored imaging protocols and multidisciplinary approaches in optimizing patient outcomes in the management of pineal region meningiomas.
